# In-depth multimodal validation of ^18^F-THK5351 for imaging monoamine oxidase-B-mediated reactive astrogliosis in Alzheimer’s and related neurodegenerative diseases

**DOI:** 10.1038/s12276-026-01757-5

**Published:** 2026-07-01

**Authors:** Heejung Chun, Wongu Youn, Hyunkeong Lim, Ryuichi Harada, Hae Young Ko, Jiyoung Yoon, Soo-Jin Oh, Philipp Seemann, Joseph G. Pepe, Brian K. Shoichet, Hye Won Lee, Shozo Furumoto, Nobuyuki Okamura, Mijin Yun, C. Justin Lee

**Affiliations:** 1 College of Pharmacy, Yonsei-SL Bigen Research Institute, Yonsei University, Incheon, Republic of Korea; 2 College of Pharmacy, Yonsei Institute of Pharmaceutical Sciences, Yonsei University, Incheon, Republic of Korea; 3 Center for Memory and Glioscience, Institute for Basic Science (IBS), Daejeon, Republic of Korea; 4 Department of Nuclear Medicine, Severance Hospital, Yonsei University College of Medicine, Seoul, Republic of Korea; 5 Division of Pharmacology, Faculty of Medicine, Tohoku Medical and Pharmaceutical University, Sendai, Japan; 6 Research Center for Accelerator and Radioisotope Science (RARiS), Tohoku University, Sendai, Japan; 7 Brain Science Institute, Korea Institute of Science and Technology (KIST), Seoul, Republic of Korea; 8 Department of Pharmaceutical Chemistry, University of California, San Francisco, CA USA; 9 Department of Internal Medicine, Institute of Gastroenterology, Yonsei University College of Medicine, Seoul, Republic of Korea

**Keywords:** Astrocyte, Translational research

## Abstract

^18^F-THK5351, initially developed as a positron-emission tomography (PET) tracer for tau pathology, was later shown to display high affinity for monoamine oxidase-B (MAO-B), raising uncertainty about the biological origin of its brain signals in neurodegenerative diseases. To resolve this ambiguity, we implemented a multi-scale validation framework integrating enzyme activity inhibition assays, molecular docking, biolayer interferometry, autoradiography, multiple transgenic and viral animal models, and human PET imaging. THK5351 selectively inhibited MAO-B while sparing MAO-A and exhibited reversible binding kinetics to recombinant MAO-B. Computational modelling localized THK5351 near the MAO-B substrate funnel, revealing moderate binding energy and weaker π–π stacking interactions compared with selective tau tracers. Autoradiographic analysis of human cortical tissue demonstrated that tracer binding was dominated by MAO-B-related signals, with a smaller contribution from tau aggregates, a difference insufficient to produce visually distinguishable patterns in clinical imaging. In APP/PS1 mice, ^18^F-THK5351 uptake colocalized with regions of reactive astrogliosis and was abolished by MAO-B inhibition, whereas overexpression of P301L-hTau induced extensive tau deposition without affecting tracer retention. MAO-B knockout reduced both tracer binding and tau phosphorylation, and viral induction of astrogliosis elevated tracer uptake that was reversed by selective MAO-B blockade. In a patient with corticobasal syndrome, tracer signals decreased during selegiline treatment and reappeared after drug withdrawal, mirroring preclinical pharmacological responses. Collectively, these findings demonstrate that ^18^F-THK5351 uptake primarily reflects MAO-B-mediated reactive astrogliosis rather than tau pathology, providing mechanistic insight into its signal origin and underscoring the value of cross-scale, multimodal validation in PET tracer development for neurodegenerative disease research.

## THK5351 Signals Linked to Monoamine Oxidase-B Not Tau Pathology

Alzheimer’s disease, a leading cause of dementia, involves complex pathological processes, including amyloid-β deposition, astrocytic reactivity and tau aggregation. This study addresses the challenge of distinguishing these processes using positron-emission tomography (PET) tracers. The authors focus on ^18^F-THK5351, initially developed as a tau tracer but later found to bind monoamine oxidase-B (MAO-B), a marker of astrocytic activity. Using enzyme assays, biolayer interferometry and molecular docking, they demonstrate that ^18^F-THK5351 primarily binds to MAO-B rather than tau. In vivo studies in mouse models and human PET imaging confirm that ^18^F-THK5351 uptake reflects MAO-B-mediated astrogliosis, with minimal tau interaction. This work provides a comprehensive validation framework for PET tracers and suggests that future research should explore the role of MAO-B in neurodegenerative diseases, potentially improving diagnostic imaging and therapeutic strategies.

This summary was initially drafted using artificial intelligence, then revised and fact-checked by the author.

## Introduction

Alzheimer’s disease (AD) remains the most prevalent cause of dementia and a major unmet medical challenge. Its pathological progression unfolds over decades — from amyloid-β (Aβ) deposition and astrocytic reactivity to tau aggregation and neuronal loss — yet the molecular relationships linking these processes remain incompletely understood. Positron-emission tomography (PET) has transformed our ability to visualize these proteinopathies in vivo, but existing tracers capture only fragments of the disease spectrum^1^. Amyloid PET provides early detection but correlates weakly with cognitive decline^2,3^, whereas modern tau tracers such as ^18^F-MK6240 (ref.^4^), ^18^F-PI2620 (ref.^5^) and ^18^F-RO-948 (ref.^6^) excel at detecting neuronal tau aggregates only at later stages^7,8^. Because astrocytic reactivity often precedes tau accumulation^9–13^, new imaging approaches are required to resolve glial and neuronal contributions within the same pathological continuum^14^.

Reactive astrocytes markedly upregulate monoamine oxidase-B (MAO-B)^15^, leading to excessive γ-aminobutyric acid (GABA) release^16–18^ and oxidative stress^19,20^ that exacerbate neurodegeneration^21^. However, reactive astrocytes often cluster near tau deposits, producing overlapping PET signals that confound the interpretation of tracer specificity^22–24^. Early-generation MAO-B tracers such as ^11^C-L-deprenyl-D_2_ (^11^C-DED) and its ^18^F-labelled analogue ^18^F-DED have been used to visualize astrocytic reactivity in vivo^25–27^; however, both exhibit significant limitations. Quantification is challenging owing to their irreversible binding kinetics, whereas the formation of radiolabelled metabolites capable of crossing the blood–brain barrier and binding to monoamine transporters further complicates signal interpretation^28–30^. In addition, their low selectivity for MAO-B over monoamine oxidase A (MAO-A) reduces target specificity^31^. Moreover, ^11^C-labelled tracers such as ^11^C-DED, ^11^C-SL25.1188 and ^11^C-BU99008 suffer from the short physical half-life of carbon-11 (≈ 20 min), requiring an on-site cyclotron and radiochemistry facility, which limits their clinical utility. These constraints have motivated the development of ^18^F-labelled, reversible and more selective MAO-B tracers that enable reliable imaging of reactive astrogliosis^32,33^.

Among these, ^18^F-THK5351 remains a unique and controversial case. Initially developed as a tau tracer, it was later shown to bind MAO-B with high affinity, blurring the interpretation of its PET signals^23,34–36^. Subsequent preclinical and clinical studies — including large-scale human imaging^37^ — revealed distinct retention patterns, even in amyloid-negative AD cases, suggesting that ^18^F-THK5351 may capture aspects of astrocytic pathology that are invisible to conventional tau tracers^23,36,38^. Autoradiography and pharmacological blocking studies have supported this dual-binding profile, showing that MAO-B inhibitors markedly reduce ^18^F-THK5351 binding, yet complete displacement is rarely achieved, implying residual affinity for tau aggregates^39,40^. However, even in cognitively normal individuals without tauopathy, the degree of tracer displacement is similar to that seen in patients with AD. More importantly, the residual affinity attributed to tau aggregates in AD is difficult to appreciate on visual analysis, and therefore even presumed tau binding is unlikely to be clinically significant^41^. Conversely, in vitro studies using recombinant tau fibrils or tau-rich tissues have reported detectable but weaker interactions relative to MAO-B, although the strength, stoichiometry and binding kinetics of these interactions remain poorly defined^23,36,38,42^. Thus, despite extensive clinical use and interpretive debate, the precise molecular determinants and relative contributions of MAO-B and tau binding to ^18^F-THK5351 PET signals have to our knowledge never been quantitatively and systematically characterized.

Here, we present a multimodal validation method that systematically dissects PET tracer–target interactions across molecular, cellular and in vivo scales. Through enzyme activity inhibition assays and biolayer interferometry, we quantify the binding of ^18^F-THK5351 to MAO-B and tau, whereas in silico protein-ligand docking and SymDOCK modelling defines its interaction on MAO-B and tau fibrils. These molecular results are integrated with autoradiography in human brain tissue, mechanistically defined mouse models and pharmacologically perturbed human PET imaging. Together, this in-depth multimodal validation confirms that ^18^F-THK5351 binds both MAO-B and tau in vitro but that its in vivo signal predominantly reflects MAO-B-mediated astrogliosis. More broadly, our study establishes a generalizable pipeline for the rigorous, multimodal validation of PET tracers that engage multiple molecular targets in neurodegenerative disease.

## Materials and methods

### Animals

APP/PS1 transgenic mice (APPswe/PSEN1dE9, JAX:004462), MAO-B knockout mice (B6; 129S-Maobtm1Shih/J, JAX:014133), C57BL/6 mice and Sprague–Dawley rats were used for in vivo experiments. Animal care followed National Institutes of Health (NIH) guidelines. The animal experimental procedures were approved by the Animal Institutional Animal Care and Use Committee of Yonsei University (Seoul, Korea). All mice were maintained in groups of 4–5/cage in the animal facility of Yonsei University (Seoul, Korea) and the Korea Institute of Science and Technology (KIST; Seoul, Korea). The animals were kept on a 12-h light–dark cycle with controlled temperature (21 ± 1 °C) and humidity (50 ± 10%) and had ad libitum access to food and water.

### Reagents

Viruses were generated from the KIST Virus Facility. The production titre was 4.5 × 10^12^genome copies per millilitre for Adeno-pGFAP-GFP and 3.5 × 10^9^ genome copies per millilitre for AAV2-EF1a-EGFP-hTau(P301L). For selegiline administration, 10 mg of r-(–)-deprenyl hydrochloride (Sigma) was dissolved in 150 ml drinking water. The dose was calculated as 10 mg/kg daily. KDS2010, a MAO-B inhibitor, was synthesized as previously described^15^. KDS2010 was administered by dissolving the compound in drinking water. The amount of KDS2010 was calculated as 10 mg/kg daily. THK-5351 and its tosylated precursor for THK-5351 (THK-5352) were custom-synthesized by Tanabe R&D Service Co. (Osaka, Japan). MK6240 was custom-synthesized by BioDuro-Sundia (Irvine, CA, USA). SMBT-1 was synthesized as previously reported^43^.

### In vitro assay of monoamine oxidase enzymatic activity and H_2_O_2_ level

Enzymatic activity of MAO-B or MAO-A was measured using an Amplex Red Monoamine Oxidase Assay Kit (Molecular Probes) according to the manufacturer’s instructions. The kit includes a 5× reaction buffer, an Amplex Red reagent, horseradish peroxidase, DMSO, H_2_O_2_, benzylamine (substrate of MAO-B) and clorgyline (inhibitor of MAO-A). After 2 h of enzyme reaction at 37 °C, hydrogen peroxide, which is produced by MAO activity, was measured by a colour change of Amplex Red reagent. The colour change was quantified by measuring absorbance at 580 nm using a microplate fluorescence reader (SpectraMax iD5, Molecular Devices).

For the reversibility test, MAO-B enzymes inhibited by each drug were dialysed using GeBAFlex-tube (Gene Bio-Application) for 3 h in a cold sodium phosphate buffer (pH = 7.4). After the dialysis, enzymes were collected and activities were measured with an MAO-B activity assay kit, respectively. MAO-B enzyme treated with DMSO was used as a negative control in each group. Enzyme activity recovery was calculated by comparing recovered enzyme activity after washing with inhibited enzyme activity before washing^44^.

### Octet biolayer interferometry

Biotinylated MAO-B (human recombinant, Sigma-Aldrich, M7441) was prepared using EZ-Link Sulfo-NHS-LC-Biotin (Thermo Fisher Scientific). Biotinylation was performed at a fivefold molar excess of reagent for 30 min at room temperature. Excess biotinylation reagent was removed by dialysis (GeBAFlex-tube, 8 kDa MWCO, Gene Bio-Application) for MAO-B, with all steps carried out at 4 °C.

Octet SSA biosensors (Sartorius) were used, and unoccupied streptavidin sites were quenched with 50 μg/ml biocytin (Thermo Fisher). Measurements were performed on an Octet R8 instrument. For MAO-B binding assays, the running buffer was PBS with 0.1% Tween-20 and 10% DMSO. The measurement sequence included baseline, protein loading, quenching, a second baseline, association and dissociation phases.

Raw signals were reference-subtracted using control sensors (without protein) and the zero-concentration condition. Steady-state analysis was performed using the Octet software to derive dissociation constants (K_D_).

### In silico molecular docking

For the in silico molecular docking study of MAO enzymes and PET tracers, structures of all the ligands mentioned (selegiline, THK5351 and SMBT-1) were built and prepared for the ligand docking in the environment of pH 7.40 ± 1 using an Epik ionizer and energistically minimized with an OPLS_2005 force field, by Ligprep module implement in the Maestro (Schrodinger LLC, NY, USA) program, respectively. X-ray crystal structure of human MAO-B bound with flavin adenine dinucleotide (FAD) (PDB code: 1GOS^45^) with a 3.0-Å resolution, and human MAO-A bound with FAD and Harmine (PDB code: 2Z5X^46^) with a 2.2-Å resolution were imported and prepared for the docking simulation in the environment of pH 7.40 ± 1, by Protein Prepare Wizard in the Maestro program, respectively. For a more accurate docking simulation, water molecules were removed and only hydrogens were minimized with an OPLS_2005 force field. Docking grids of 36 Å×36 Å×36 Å were set, and docking study was performed within the grids using Glide module in the Maestro program, with the standard precision scoring function. Along the grids, we chose the grid that includes most-likely docking sites, checked by lowest docking score, and already known binding sites from previous studies^47,48^. Molecular mechanics with generalized Born and surface area solvation method^49^ with an OPLS_2005 force field and VSGB solvation model was used to calculate ligand binding energies, with a previously simulated ligand docking model, respectively. Because we assumed the protein structure to be fixed and only ligand conformation change was considered, binding free energies cannot represent the whole free energies from molecular docking. Also, docking score is the arbitrary value used to compare the binding properties; thus, the absolute values are not for use in the quantitative analysis but are recommended for use in relative comparisons between each ligand.

### Symmetry docking of MK6240, THK5351 and SMBT-1 to tau fibrils (SymDOCK)

For molecular docking employing SymDOCK, the paired helical filament tau fibril cryo-EM structure (PDB: 8UQ7) in complex with the PET-tracer MK6240 was used^50^. The fibril was protonated by means of Schrödinger’s Maestro protein preparation pipeline (physiological pH 7.4 ± 1). A total of 45 binding hot spots (spheres)^51,52^ were used based on the binding pose of the respective complexed ligand (MK6240) of the fibrils. Molecular docking grids used for determining the energy contributions of each term in the DOCK3.8 scoring function were precalculated using a grid-based version of the AMBER force-field for the van der Waals component, and were calculated using the Poisson–Boltzmann method QNIFFT for the electrostatics component^53–55^. Context-dependent ligand desolvation grids were generated via an adapted version of the generalized Born method^56^. For molecular docking of ligands in a stacked fashion, the recently developed SymDOCK was applied as described^57^. SymDOCK is an extension to UCSF DOCK and adds a ligand–ligand van der Waals energy score to evaluate the energy contribution of stacked ligands. Ligands (MK6240, THK5351, SMBT-1) were prepared as described for molecules in the ZINC library^58^. Root mean square deviation (RMSD) calculations to compare the experimental and docked poses of MK6240 were carried out by means of UCSF Chimera^59^.

### Stereotaxic surgery

Mice were anesthetized with vaporized isoflurane and placed in a stereotaxic frame (Kopf Instruments). Adeno-associated virus for hTau-P301L overexpression (AAV2-EF1a-EGFP-hTau(P301L)) was injected using a stereotaxic micro-injector (Stoelting Co.). Virus (1:3 diluted with saline, total 2 μl) was injected into the molecular layer of left CA1 (dorsal hippocampus, anterior–posterior (AP): −1.8 mm, medial–lateral (ML): +1.45 mm, dorsal–ventral (DV): −1.75 mm using a micro-syringe pump (Micro 4, WPI, USA) with a 33-gauge needle (WPI, USA) (0.2 μl/min). Neuropathological experiments were performed 3–4 months after injection.

### Immunofluorescence staining and confocal imaging

Mice were deeply anesthetized with 2% avertin (20 mg/g, intraperitoneally) and perfused with 0.9% saline followed by ice-cold 4% paraformaldehyde. Excised brains were post-fixed overnight at 4 °C in 4% paraformaldehyde and dehydrated in 30% sucrose for 48 h. Coronal hippocampal sections were cut at 30 μm in a cryostat and stored in storage solution at 4 °C. Sections from storage solution were washed in PBS and incubated for 1 h in a blocking solution (0.3% Triton X-100, 2% donkey serum in 0.1 M of PBS). Primary antibodies in blocking solution were immunostained on a shaker at 4 °C overnight. After washing in PBS three times, sections were incubated with corresponding fluorescent secondary antibodies for 2 h at room temperature and then washed with PBS three times. DAPI staining was done by adding DAPI solution (1:5,000; Pierce) during the second washing step. Finally, sections were mounted with fluorescent mounting medium (Dako) and dried. A series of fluorescent images were obtained with a ZEISS confocal microscope (26-μm Z stack images in 2-μm steps were processed using ImageJ software (NIH). Primary antibodies were diluted to the following amounts: chicken anti-GFAP (Abcam, AB5541, 1:500); rabbit anti-MAO-B (Abcam, ab137778, 1:300); mouse anti-pTau (AT8) (Thermo Fisher Scientific, MN1020, 1:500); rabbit anti-pTau (S396) (Abcam, ab109390, 1:500); rabbit anti-pTau (S199) (Abcam, ab81268, 1:500); mouse anti-NeuN (Sigma-Aldrich, MAB377, 1:1000); anti-NFT (Sigma-Aldrich, ab1518, 1:500). Secondary antibodies (donkey anti-mouse anti-IgG (647) (Invitrogen, A31571), donkey anti-rabbit anti-IgG (594) (Invitrogen, A21207), donkey-anti-chicken anti-IgG (405) (Jacksonimmuno, 703-475-155) and donkey anti-chicken anti-IgG (488) (Invitrogen, A78948) were diluted 1:500 in the blocking solution.

### Micro-positron emission tomography imaging

Micro-PET imaging was performed using an Inveon PET scanner (Siemens Medical Solutions, USA). Animal cohorts were divided into three experimental sets: (1) APP/PS1 transgenic mice (*n* = 10, with and without selegiline administration) and C57BL/6 wild-type (WT) mice (*n* = 5); (2) MAO-B knockout mice (*n* = 5) and their corresponding WT mice (*n* = 4); and (3) Sprague–Dawley rats, which were assigned to vehicle (*n* = 6) and KDS2010 (*n* = 4) groups. Animals were anaesthetized with 2.5% isoflurane before the administration of radiotracer.

A dose of 17.76 ~ 22.76 MBq (480 ~ 615 µCi/200 µl in saline) of ^18^F-THK5351, adjusted according to the body weight of the mice, was injected into the tail vein. After a 40-min uptake period, mice were sacrificed by cardiac perfusion, and the brain tissue was rapidly isolated for subsequent experiments. ^18^F-THK5351 PET data were acquired for 60 min using the isolated brain tissue from the mice. This protocol was supported by our preliminary dynamic study of ^18^F-THK5351 in the control and adenovirus-injected mice, demonstrating that ^18^F-THK5351 level is well stabilized between 30 and 40 min after injection (data not shown).

For the ^18^F-THK5351 PET scan, the Adeno-pGFAP-GFP virus-injected rat model without (control group, *n* = 6) and with KDS2010 treatments (*n* = 4) were administered 20 MBq (0.54 mCi/200 µl in saline) of ^18^F-THK5351. Rats were anaesthetized with isoflurane (2.5% in 100% oxygen), and maintained on a heating pad for 40 min to allow for sufficient uptake. Subsequently, in vivo ^18^F-THK5351 PET imaging of the rat brain was performed for 40 min.

All PET data were reconstructed with 3D ordered subset expectation-maximization with two iterations and 18 subsets. The voxel size was 0.385 × 0.385 × 0.796 mm^3^ and the matrix size was 256 × 256 × 159 mm^3^. Micro-PET image processing was performed using the Analysis of Functional NeuroImages software. ^18^F-THK5351 PET images were registered to the MRI template of Sprague–Dawley rat brain using an automated template-based registration algorithm and manual manipulation for minor misalignment. To statistically compare ^18^F-THK5351 PET images in the Adeno-pGFAP-GFP virus-injected models (with and without KDS2010 treatment), a voxel-wise *t* test was performed using 3dttest in Analysis of Functional NeuroImages software (statistical threshold, *P* < 0.01). The 3D rendering images fused a T1-weighted MRI with the statistical parametric map and were displayed using MRIcroGL software (https://www.mccauslandcenter.sc.edu/mricrogl/).

### Autoradiography experiment

Human brain tissues were obtained from Tohoku University. The procedures were reviewed by the Ethics Committee of the Graduate School of Medicine, Tohoku University. Non-fixed frozen sections (12 μm) were generated by a cryostat. The brain sections were dried and dipped in PBS for a total of 25 min, and then pre-incubated in PBS containing 1% bovine serum albumin (BSA). Brain sections were then incubated for 30 min at room temperature with 3 nM ^3^H-THK5351 (molar activity, 2.96 TBq/mmol; radiochemical purity, 98.9%, Sekisui Medical Inc., Tokyo, Japan) or 0.2–1.2 nM ^18^F-THK5351 (molar activity, 446 GBq/μmol; radiochemical purity, 99%, produced at the Research Center for Accelerator and Radioisotope Science of Tohoku University). After incubation, sections were washed sequentially with PBS containing 1% BSA for 5 min, followed by PBS for 5 min twice. Dried sections were exposed to an imaging plate for tritium (BAS IP TR 2025 E, Cytiva, UK) for 3 days and an imaging plate for fluorine-18 (BAS IP MS 2025 E). The autoradiographic images were obtained from Typhoon FLA-7000 (GE Healthcare) and Typhoon FLA-9000 (GE Healthcare). In vitro competitive autoradiography was performed in the presence of 3 μM unlabelled ligands or inhibitors, which were obtained from Sigma-Aldrich and Tocris Bioscience (Bristol, UK). Serial sections were post-fixed in 4% paraformaldehyde for 30 min and immunostained by anti-tau (AT8), anti-Aβ (6 F/3D; Dako, Santa Clara, CA, USA) and anti-MAO-B (HPA002328l Sigma-Aldrich, St Louis, MO, USA) antibodies. Gallyas-Braak silver staining was also performed in the AD brain section.

### In vitro competitive binding experiment

The reaction mixture contained ^3^H-MK6240 (1.5 nM; specific activity, 0.83 TBq/mmol; radiochemical purity, 99%; ViTrax, Placentia, CA, USA) and brain homogenates (0.5 μg), in a final volume of 200 μl. Non-specific binding was defined in the presence of 100 nM unlabelled MK6240. The mixture was incubated at room temperature for 3 h, and separation of bound from free radioactivity was achieved by filtration under reduced pressure (Multiscreen HTS Vacuum Manifold, Multiscreen HTS 96-well 0.65 μm filtration plate; Millipore, Billerica, MA, USA), followed by three washes with PBS containing 0.1% BSA. The filters were incubated in 2 ml scintillation fluid (Emulsifier-Safe; Perkin Elmer, Boston, MA, USA), and a β counter (LS6500 liquid scintillation counter; Beckman Coulter, Brea, CA, USA) was used to measure the radioactivity.

### Human positron-emission tomography imaging

Five patients, diagnosed with AD, were enrolled from the dementia outpatient clinic at Severance Hospital, Yonsei University Health System. AD diagnosis was confirmed based on the criteria set forth by the National Institute of Neurological and Communicative Disorders and Stroke, in conjunction with the Alzheimer’s Disease and Related Disorders Association (NINCDS-ADRDA), and the guidelines proposed by Petersen et al.^60^. Eight healthy controls, with no prior history of neurological disorders or subjective cognitive impairment, were recruited. In the study observing MAO-B-dependent PET imaging in CBS, one patient was selected.

All participants underwent the Seoul Neuropsychological Screening Battery (SNSB), Brain MRI and ^18^F-FDG, ^18^F-florbetaben (FBB) and ^18^F-THK5351 PET. This study was approved by the institutional review board of Severance Hospital (IRB No. 4-2016-0411), and written informed consent was obtained from all participants. All patients fasted at least 6 h prior to ^18^F-FDG PET scans, which were performed using Discovery 600 (General Electric Healthcare). For the ^18^F-FDG PET scan, 4.1 MBq (0.11 mCi) per body weight (kg) of ^18^F-FDG was intravenously administered to the patients.^18^F-FBB was injected at a dose of 300 MBq (8.1 mCi). A dose of 185 MBq (5 mCi) of ^18^F-THK5351 was intravenously administered to the patients. After 40 min for ^18^F-FDG, 90 min for ^18^F-FBB, 60 min of uptake period for ^18^F-THK5351 and PET scans were performed. The scan time of ^18^F-FDG, ^18^F-FBB and ^18^F-THK5351 were 15 min, 20 min and 20 min, respectively. The information on the CT scan for attenuation correction was as follows: 0.5-s rotation time, 200 mA, 120 kVp, 3.75-mm section thickness, 10.0-mm collimation, and 9.375-mm table feed per rotation. The acquired PET data were reconstructed using the ordered subset expectation-maximization with two iterations and 32 subsets.

The PET images of 8 normal control subjects and 5 patients with AD, acquired for both ^18^F-FDG and ^18^F-THK5351, underwent co-registration and normalization processes using SPM. Subsequently, regions exhibiting differences between the patient and control groups were extracted through statistical analysis using a two-sample *t* test.

The patient with CBS underwent an initial ^18^F-THK5351 PET scan 10 months following the commencement of selegiline treatment. A second ^18^F-THK5351 PET scan was performed 8 months after drug discontinuation to assess changes in the brain associated with selegiline cessation.

### Statistical analysis

All experiments in this study are representative of at least two biological replicates. For the APP/ PS1 and MAO-B KO mice, mice with the required genotypes were randomly assigned to the experimental groups and treated in the same way. Data distribution was assumed to be normal, but this was not formally tested. Data are presented as the mean ± SEM. All statistical analyses were performed using Prism v.8.4.3 (GraphPad Software); an unpaired, two-tailed Student’s *t* test was used to compare two groups. A one-way analysis of variance (ANOVA) with Tukey’s multiple comparisons test was used to compare more than two groups, as indicated in the figure legends. Statistical differences were considered significant when *P* < 0.05 and are indicated in the figures or figure legends. No statistical methods were used to predetermine sample sizes, but sample sizes are similar to those reported in previous publications.

### Ethics approval and consent to participate

All animal experimental procedures were approved by the Animal Institutional Animal Care and Use Committee of Yonsei University (Seoul, Korea). Human brain tissues were obtained from Tohoku University, and the procedures were reviewed by the Ethics Committee of the Graduate School of Medicine, Tohoku University. Human PET study was approved by the institutional review board of Severance Hospital (IRB No. 4-2016-0411), and written informed consent was obtained from all participants.

## Results

### Selective and partially reversible inhibition of monoamine oxidase-B by THK5351 in vitro

MAOs are mitochondrial flavoproteins consisting of two isoforms, MAO-A and MAO-B, which share high sequence similarity but differ in substrate preference and active-site architecture. Although both catalyse oxidative deamination of monoamines, MAO-A primarily metabolizes serotonin, whereas MAO-B preferentially targets phenylethylamine and is highly expressed in astrocytes, enabling the development of selective inhibitors for each isoform^61^.

**Fig. 1:**
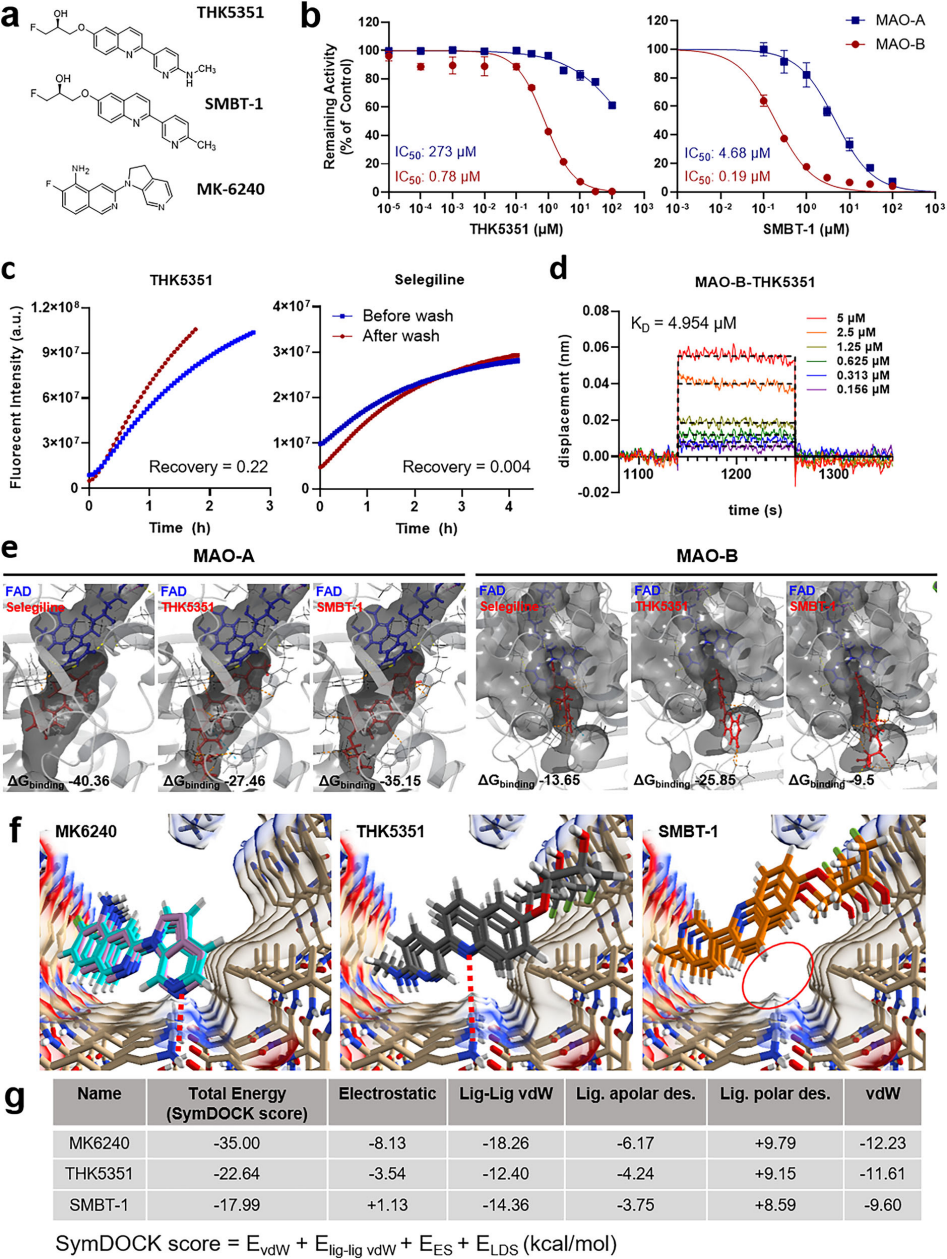
Comparative binding properties of THK5351 to monoamine oxidase-A, monoamine oxidase-B and tau aggregates. **a** Chemical structures of the positron-emission tomography (PET) tracers used. **b** Dose-dependent response curves of PET tracers in monoamine oxidase (MAO)-B and MAO-A enzyme assays. **c** MAO-B reversibility test traces of THK5351 (left) and selegiline (right). **d** Octet measurement traces of MAO-B-THK5351. **e** Structures of (left) flavin adenine dinucleotide (FAD)–MAO-A enzyme complex, and (right) FAD–MAO-B enzyme complex, and its binding site for the PET tracers (red), respectively. To compare the binding sites of FAD and each ligands, FAD molecule was represented as blue in every model structure. Binding Gibbs free energy was calculated with the molecular mechanics with generalized Born and surface area solvation method for each pose. **f** SymDOCK docking of PET-tracers MK6240, THK5351 and SMBT-1 to PHF-tau. View down the tau-fibril, poses of ligands are represented as a stack. The experimental cryo-EM pose of MK6240 (plum) in PHF-tau (PDB: 8uq7, beige) and the docked pose of MK6240 (cyan). The docked pose superposes well to the experimental structure, with a root mean square deviation of 0.6 Å. MK6240 is in close proximity to Lys353, establishing a hydrogen bond with a distance of 2.6 Å (red dotted line) (left). View down the tau fibril. THK5351 (grey) docked pose in PHF-tau (beige). THK5351 fails to establish a hydrogen bond with the target fibril (Lys353) and thus has reduced electrostatic complementarity, as measured by its electrostatic energy score. The distance between the Lys353 nitrogen and the pyridine nitrogen of THK5351 is 5.69 Å (red dotted line), too far to establish a hydrogen bond (middle). Docked pose of SMBT-1 (orange). SMBT-1 performs even worse than THK5351 and MK6240. The ligand fails to accommodate the pocket (red circle) and also fails to establish meaningful electrostatic interactions with the target. The electrostatics are even penalized (+ 1.13 kcal/mol), and the overall total energy score is weaker too. **g** SymDOCK energy scores. The total energy score is the sum of the van der Waals, ligand–ligand van der Waals energy (unique to SymDOCK), electrostatic and ligand desolvation scores (apolar and polar). Data are presented as the mean ± SEM.

To assess whether THK5351 directly interacts with these enzymes, we first performed enzyme activity assays using purified recombinant proteins (Fig. 1a, b). THK5351 inhibited MAO-B activity in a concentration-dependent manner with an IC_50_ of 780 nM, whereas a very weak inhibitory effect was detected for MAO-A (IC_50_ of 273 μM, Fig. 1b). Because the MAO-B assay relies on quantification of H_2_O_2_ generated during catalysis (Supplementary Fig. 1a), we tested whether THK5351 might interfere by scavenging H_2_O_2_ or interacting with horseradish peroxidase (HRP). An H_2_O_2_ assay showed no such effect (Supplementary Fig. 1b), confirming that THK5351 directly inhibits the MAO-B enzyme. For comparison, we evaluated another radiotracer: SMBT-1, a selective MAO-B PET ligand (Fig. 1a). SMBT-1 potently inhibited MAO-B activity (IC_50_ = 0.19 μM) but also inhibited MAO-A at higher concentrations (IC_50_ = 4.68 μM), reflecting greater potency but lower selectivity relative to THK5351 (Fig. 1b).

Next, we evaluated the reversibility of THK5351 using a dialysis-based enzyme reversibility assay. MAO-B was first pre-incubated with THK5351, followed by dialysis to remove unbound compound, and residual enzyme activity was subsequently quantified. Selegiline and DMSO were included as irreversible and vehicle controls, respectively. As expected, selegiline exhibited minimal recovery of enzymatic activity after dialysis (0.4%, Fig. 1c), consistent with irreversible inhibition. In contrast, THK5351 showed partial recovery of MAO-B activity (22%, Fig. 1c), indicating a partially and slowly reversible mode of inhibition under the conditions tested.

### Direct binding of THK5351 to monoamine oxidase-B by biolayer interferometry

We next assessed whether THK5351 directly binds to MAO-B using biolayer interferometry (BLI), which enables real-time, label-free measurement of binding kinetics and is well suited to comparative ligand screening. Biotinylated MAO-B proteins were immobilized on streptavidin-coated sensors, and baseline signals were recorded before exposure to tracer solutions across a concentration gradient. Association and dissociation phases were then monitored to derive binding kinetics. THK5351 demonstrated direct binding to MAO-B with a K_D_ of 4.95 μM, consistent with its IC_50_ value from the enzyme assay (0.78 μM) (Fig. 1d).

### In silico docking supports monoamine oxidase-B binding of THK5351, and less binding towards tau fibril

To complement the enzyme assays, we performed molecular docking simulations of THK5351 and comparator tracers with MAO-A and MAO-B. Docking was carried out with the essential cofactor FAD bound within the enzymes (Fig. 1e), and Gibbs free energy change (ΔG_binding_) was calculated using the Molecular mechanics with generalized Born and surface area solvation method^49^. When docked into the FAD-bound enzymes, all tracers localized near the known selegiline binding pocket, adjacent to the substrate funnel below the FAD binding site. Consistent with the enzyme assays, THK5351 showed relatively weak interaction energies in the MAO-A–FAD complex, aligning with its lack of MAO-A inhibition, whereas SMBT-1 docked more stably to MAO-A, consistent with its stronger inhibitory effect. In contrast, THK5351 showed stronger ΔG_binding_ in the MAO-B active site than selegiline or SMBT-1, supporting the higher possibility of direct interaction within the substrate pocket of MAO-B.

To examine ligand–tau interactions at the fibrillar level, we performed symmetric molecular docking (SymDOCK) simulations using the PHF tau structure (PDB: 8UQ7)^57^. PET ligands, despite their chemical simplicity and limited opportunities for hydrogen bonding, often bind amyloid fibrils with nanomolar or even subnanomolar affinity. Structural analyses have revealed that this high affinity largely arises from ligand stacking along the fibril axis, where ligand–ligand van der Waals interactions dominate over direct fibril–ligand contacts. SymDOCK is specifically designed to model such stacking interactions by extending the DOCK 3.8 platform to include affine transformations of fibril monomers and ligand–ligand van der Waals energy terms.

As a validation, MK6240 was re-docked into its experimental cryo-EM binding site, yielding excellent pose reproduction (best RMSD = 0.6 Å; Fig. 1f). When applied to THK5351 and SMBT-1, the results revealed distinct binding behaviours. THK5351 produced a weaker overall SymDOCK score (–22.6 kcal/mol) than MK6240 (–35.0 kcal/mol), mainly because of reduced ligand–ligand van der Waals stabilization and loss of electrostatic interactions (Fig. 1g). Structural inspection showed that THK5351 lacks the critical hydrogen bond with Lys353 observed in MK6240; the quinoline nitrogen lies too distant to serve as an effective acceptor (Fig. 1f). SMBT-1 yielded the least favourable score (–18.0 kcal/mol) and lacked key hydrogen-bonding features (Fig. 1g). These findings suggest that although THK5351 can occupy the PHF-tau binding groove, its weaker stacking and fewer intermolecular interactions likely explain its reduced tau-binding affinity relative to MK6240.

### Characterization of THK5351 binding in postmortem human brain

**Fig. 2:**
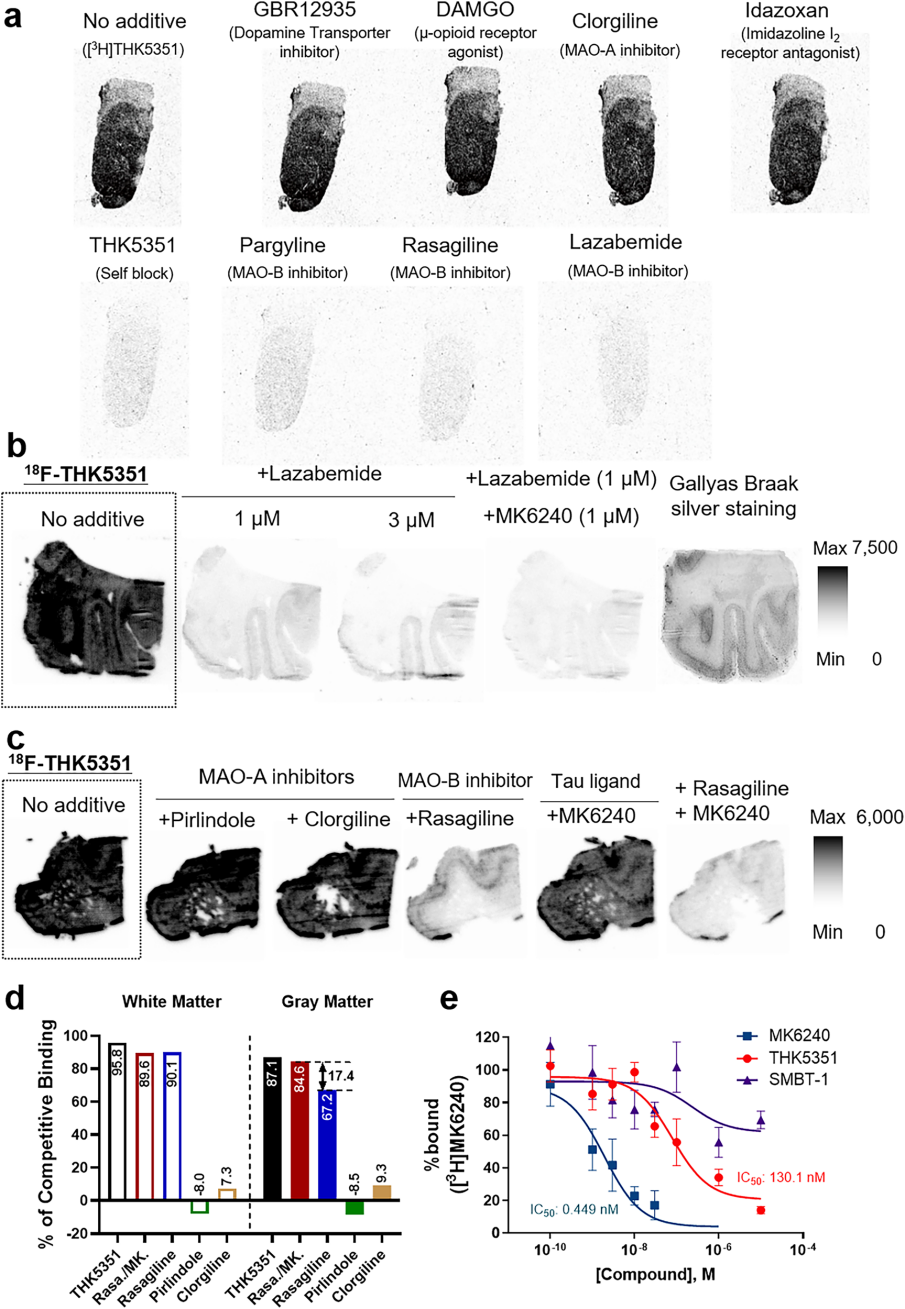
Comparative binding properties of THK5351 in human brain tissue. **a** Autoradiography images of ^3^H-THK5351 with competitive ligands such as THK5351 (self block), GBR12935 (dopamine transporter inhibitor), DAMGO (mu-opioid receptor agonist), clorgiline (MAO-A inhibitor), lazabemide (MAO-B inhibitor), idazoxan (imidazoline I2 receptor antagonist), pargyline and rasagiline (MAO-B inhibitors). **b** Autoradiography images of ^18^F-THK5351 with lazabemide (1 μM or 3 μM) and MK6240 (1 μM), and Gallyas Braak silver staining image in human Alzheimer’s disease brain tissue. **c** Autoradiography images of ^18^F-THK5351 with competitive ligands in human Alzheimer’s disease brain tissue. **d** Quantification of displacement by MAO-B inhibitor (rasagiline), tau tracer (MK6240) or MAO-A inhibitor (pirlindole or clorgiline) in ^18^F-THK5351 binding. The signal ratio of tau and MAO-B was calculated 1:3.86. **e** Competition assay of ^3^H-MK6240 (1.5 nM) with THK5351 and SMBT-1. The IC_50_ values of MK6240 and THK5351 are 0.449 nM and 130.1 nM, respectively. Data are presented as the mean ± SEM.

We next characterized THK5351 binding in postmortem human brain tissue using competitive autoradiography. In basal ganglia from controls without dementia, where MAO-B is constitutively expressed, we observed strong ^3^H-THK5351 binding (Fig. 2a). This binding was almost completely displaced by MAO-B inhibitors, including pargyline, rasagiline and lazabemide, but was unaffected by ligands targeting MAO-A (clorgiline), μ-opioid receptors (DAMGO), dopamine transporters (GBR12935) or imidazoline I₂ receptors (idazoxan) (Fig. 2a). Consistent with these pharmacological profiles, immunohistochemistry of adjacent sections revealed strong MAO-B immunoreactivity, whereas phosphorylated tau and Aβ were largely undetectable in these basal ganglia samples (Supplementary Fig. 2a). These data confirm that in the absence of tau pathology, THK5351 binding in human brain tissue predominantly reflects MAO-B.

We then examined AD brain tissue containing abundant tau pathology. Autoradiography demonstrated robust ^18^F-THK5351 binding in both grey and white matter, with binding displaced by lazabemide in a dose-dependent manner (Fig. 2b). Notably, residual laminar binding in cortical grey matter persisted even at 3 μM lazabemide but was further reduced by cold MK6240, suggesting that THK5351 exhibits weak tau binding in addition to its major MAO-B interaction. Silver staining confirmed the presence of neurofibrillary tangles in these regions (Fig. 2b).

To further evaluate the target selectivity of ^18^F-THK5351, autoradiography was performed in cortical sections from AD brains in the presence of inhibitors targeting MAO-A, MAO-B or tau. In white-matter regions devoid of tau aggregates, ^18^F-THK5351 binding was almost completely abolished by the MAO-B inhibitor rasagiline, whereas MAO-A inhibitors (pirlindole or clorgiline) had no effect (Fig. 2c). In grey-matter regions, rasagiline substantially reduced but did not fully eliminate the signal, whereas the combined application of rasagiline and the tau tracer MK6240 completely displaced the residual binding (Fig. 2c, Supplementary Fig. 2b). Quantitative analysis confirmed that in white matter, rasagiline alone displaced >95% of the tracer signal, consistent with the absence of tau pathology. In grey matter, rasagiline alone reduced binding to 67.2%, and the combination with MK6240 further reduced to an extent of 84.6%, corresponding to an additional 17.4% displacement (Fig. 2d). These findings suggest that approximately three-quarters of the ^18^F-THK5351 signal in cortical grey matter arises from MAO-B binding, whereas only one-quarter originates from weak interactions with tau aggregates. Furthermore, MAO-A inhibitors produced only negligible displacement in both grey and white matter, consistent with their lack of involvement in THK5351 binding (Fig. 2d). Supporting this, competition assays using ^3^H-MK6240 showed dose-dependent displacement by THK5351, though with a markedly weaker affinity (IC₅₀ = 130 nM) than MK6240 itself (0.449 nM), representing an ~290-fold difference (Fig. 2e). Together, these results indicate that THK5351 binding in human brain tissue is primarily mediated by MAO-B, with additional but weaker binding to tau in AD cortex, and negligible interaction with MAO-A.

### ¹⁸F-THK5351 uptake in APP/PS1 mice reflects monoamine oxidase-B elevation rather than tau pathology

**Fig. 3:**
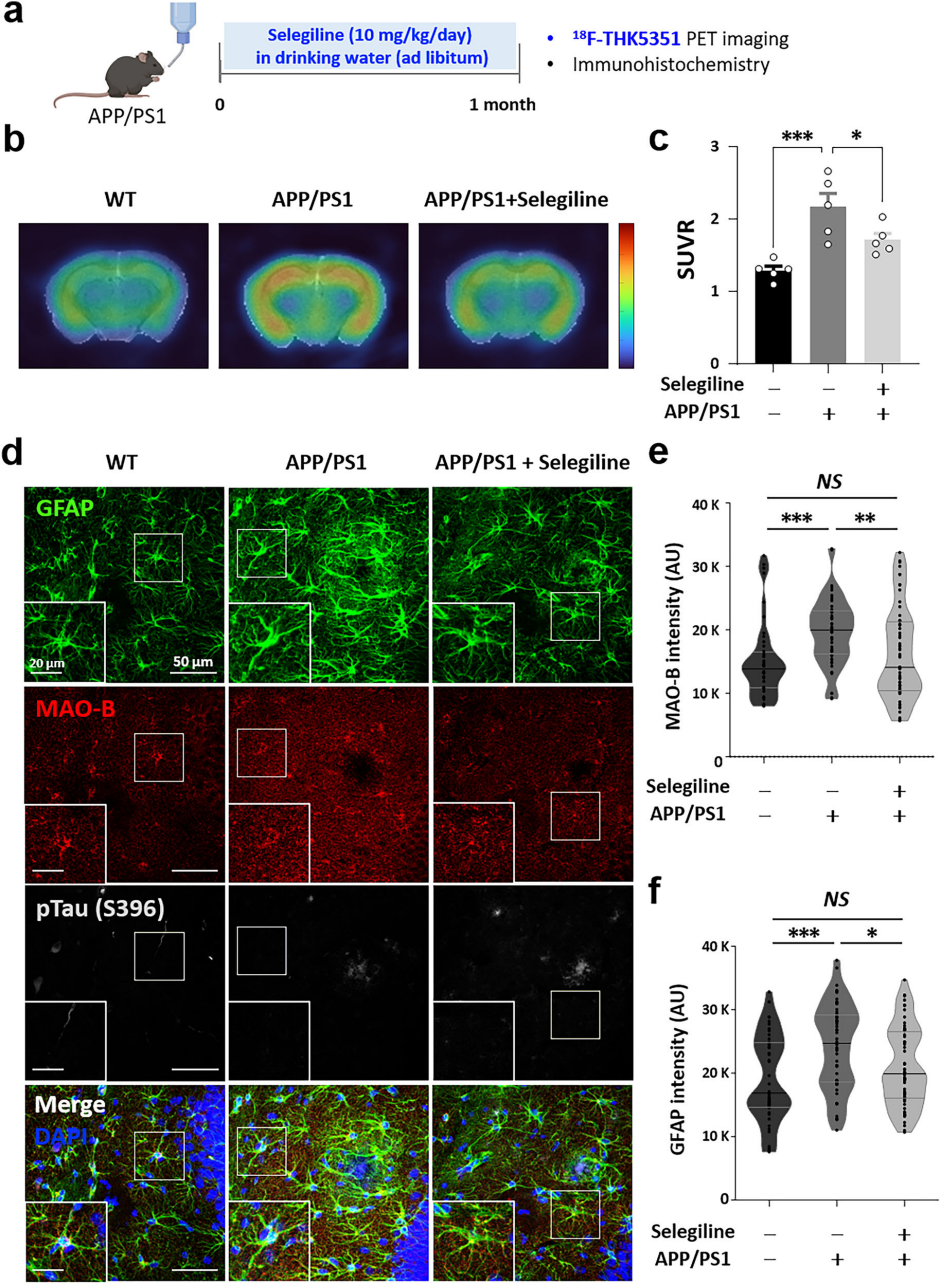
Monoamine oxidase-B-mediated ^18^F-THK5351 positron-emission tomography signals in APP/PS1 mice. **a** Schematic timeline of experiment with selegiline treatment in APP/PS1 mice. **b** Representative micro-positron-emission tomography (PET) images of ^18^F-THK5351 in selegiline-treated APP/PS1 mice. **c** Standardized uptake value ratio (SUVR) of ^18^F-THK5351 signals. **d** Representative immunohistochemistry images for GFAP, monoamine oxidase (MAO)-B and pTau (S396) in selegiline-treated APP/PS1 mice brains. **e** Mean intensity of MAO-B in selegiline-treated APP/PS1 mice brains. **f** Mean intensity of GFAP in selegiline-treated APP/PS1 mice. Data are presented as the mean ± SEM (c) or shown as violin plots with individual points, median, and interquartile range (25th–75th) indicated (e, f). *P < 0.05, **P < 0.01, ***P < 0.001, NS, non-significant.

To investigate whether the in vitro MAO-B binding of THK5351 is evident in vivo, we examined ^18^F-THK5351 uptake in APP/PS1 transgenic mice, a well-established amyloidogenic model that develops reactive astrogliosis and elevated MAO-B expression around amyloid plaques but does not form mature neurofibrillary tangles (NFTs)^16,62^ (Fig. 3a). ^18^F-THK5351 PET showed increased uptake in the hippocampus of APP/PS1 mice compared with WT controls (Fig. 3b, c). To test whether this increase reflected MAO-B activity, we treated APP/PS1 mice with the irreversible MAO-B inhibitor selegiline for 1 month (Fig. 3a). Immunohistochemistry confirmed elevated MAO-B and glial fibrillary acidic protein (GFAP) expression in hippocampal CA1 regions of APP/PS1 mice, which was reduced by selegiline treatment (Fig. 3d–f and Supplementary Fig. 3). Correspondingly, ^18^F-THK5351 uptake was significantly decreased in selegiline-treated mice compared with untreated APP/PS1 mice (Fig. 3b, c).

Notably, phosphorylated tau (Ser396) was not detected in WT, APP/PS1, or selegiline-treated APP/PS1 brains (Fig. 3d), as well as other forms of phosphorylated tau (Supplementary Fig. 3) consistent with the absence of tau tangles in this model. Thus, the elevated ^18^F-THK5351 uptake observed in APP/PS1 mice is attributable to increased MAO-B expression in reactive astrocytes, and not to pathological tau proteins.

### ¹⁸F-THK5351 uptake is minimally influenced by overexpression of P301L-hTau

**Fig. 4:**
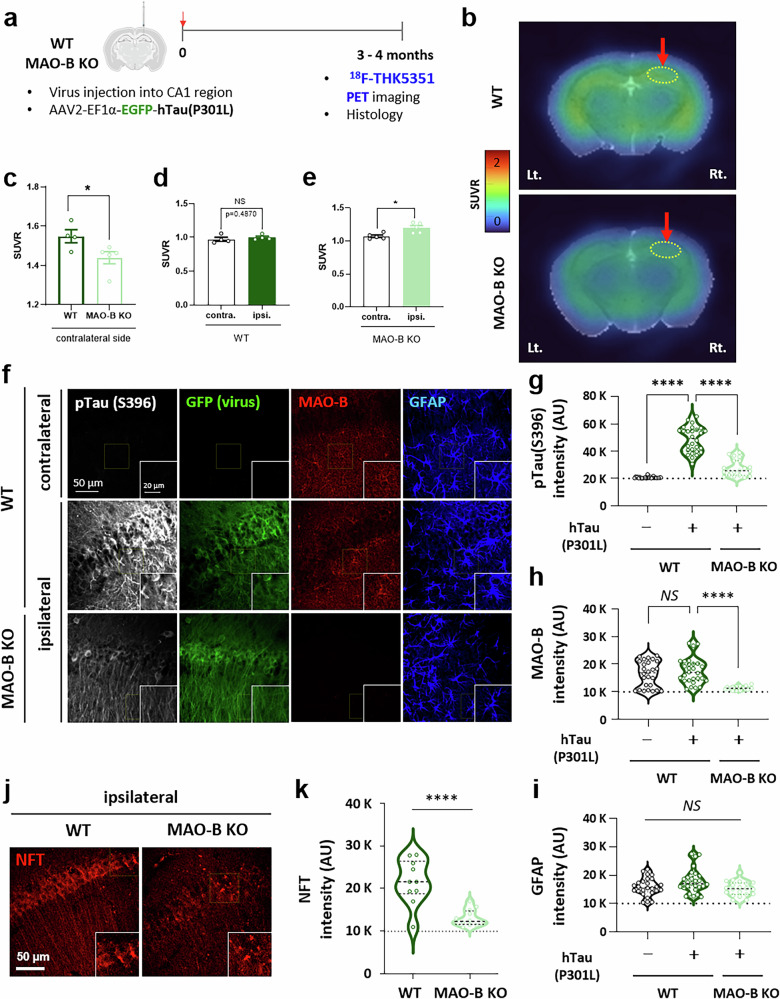
Monoamine oxidase-B-dependent ^18^F-THK5351 positron-emission tomography signals and neurofibrillary tangle formation in hTauP301L-overexpressed mice. **a** Schematic timeline of an experiment with virus-mediated hTauP301L overexpression in wild type (WT) and monoamine oxidase (MAO)-B knockout (KO) mice. **b** Representative micro-positron-emission tomography (PET) images of ^18^F-THK5351 in hTauP301L-overexpressed WT and MAO-B KO mice. **c–e** Standardized uptake value ratio (SUVR) of ^18^F-THK5351 signals. **f** Representative immunohistochemistry images of GFAP, MAO-B and pTau (S396) in hTauP301L-overexpressed mice. **g–i** Mean intensity of pTau(S396) (**g**), MAO-B (**h**) and GFAP (**i**) in hTauP301L-overexpressed WT or MAO-B KO mice. **j** Representative immunohistochemistry images of neurofibrillary tangles in hTauP301L-overexpressed mice. **k** Intensity of neurofibrillary tangles in hTauP301L-overexpressed mice. Data are presented as the mean ± SEM (**c–e**), or shown as violin plots with individual points, median and interquartile range (25th–75th) indicated. *P < 0.05, ****P < 0.0001. NS, non-significant.

To dissect the relative contributions of tau pathology and MAO-B activity to ^18^F-THK5351 uptake, we used an adeno-associated virus (AAV)-based mouse model locally overexpressing human 4 R tau carrying the P301L mutation (AAV-EF1α-EGFP-hTau(P301L)) in the hippocampus (Fig. 4a). This model offers a controlled system for inducing localized and quantifiable tau pathology, allowing direct comparison between ipsilateral and contralateral hemispheres within the same animal. In WT mice, the injected hippocampus displayed strong accumulation of phosphorylated tau (Ser396) and neurofibrillary tangle-like aggregates relative to the contralateral side (Fig. 4f, g and Supplementary Fig. 4). However, ^18^F-THK5351 PET uptake did not differ between hemispheres (Fig. 4b–d), indicating that tau aggregation alone does not substantially alter tracer retention in vivo. Immunostaining confirmed that MAO-B and GFAP levels remained unchanged after P301L-hTau overexpression (Fig. 4f–i).

To further probe the mechanistic link between MAO-B and tau pathology, we examined MAO-B knockout (KO) mice injected with the same AAV-hTau(P301L) vector. In contralateral regions, ^18^F-THK5351 uptake was greatly reduced in KO mice compared with WT controls (Fig. 4c), consistent with MAO-B being the dominant molecular target of the tracer. Within the injected hippocampus, a slight ipsilateral increase in uptake was observed, suggesting that, in the absence of MAO-B, P301L-hTau overexpression can contribute minimally to tracer binding (Fig. 4e). Interestingly, phosphorylated tau and tangle-like inclusions were markedly reduced in the hippocampus of MAO-B KO mice than in that of WT mice (Fig. 4f–k), implicating MAO-B activity as a potential modulator of tau aggregation. Collectively, these findings demonstrate that MAO-B is the principal determinant of ^18^F-THK5351 uptake in vivo and may also influence tau pathology.

### ^18^F-THK5351 detects monoamine oxidase-B-mediated reactive astrogliosis independent of proteinopathy

**Fig. 5:**
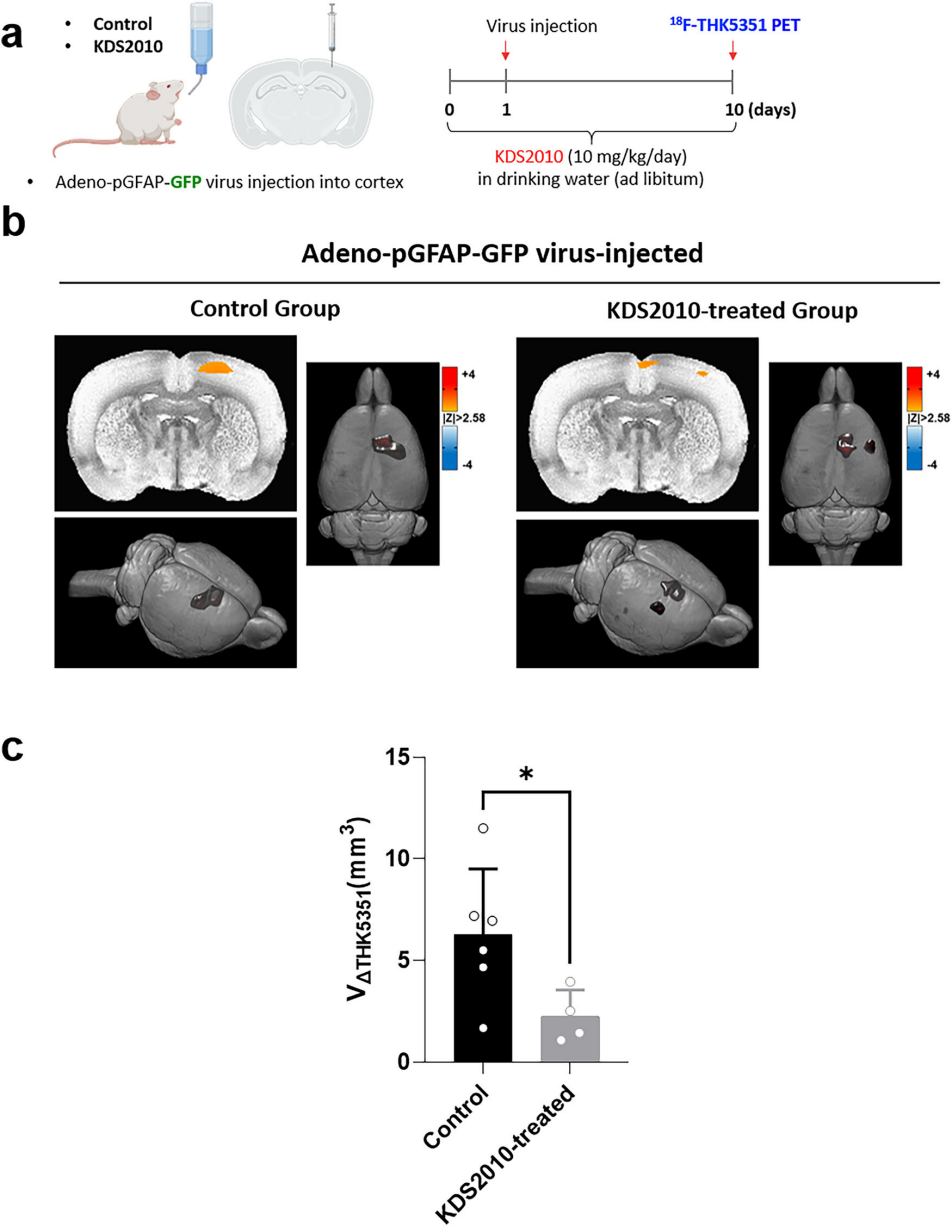
Monoamine oxidase-B-mediated ^18^F-THK5351 positron-emission tomography signals in an adenovirus-induced reactive astrogliosis model. **a** Schematic description of adenovirus-induced reactive astrogliosis model. **b** ^18^F-THK5351 positron-emission tomography (PET) images in Adeno-pGFAP-GFP-injected rat model with KDS2010 administration. **c** Quantification of the volume of increased ^18^F-THK5351 uptake (*n* = 6 and 4 rats for the vehicle and KDS2010 groups, respectively). Data are presented as the mean ± SEM. *P < 0.05.

To determine whether ^18^F-THK5351 can visualize reactive astrogliosis independently of amyloid or tau pathology, we utilized an adenoviral model that induces robust astrocytic activation and neuroinflammation in the absence of protein aggregates (Fig. 5a). This model has previously been used to elevate astrocytic MAO-B expression without tau aggregate expression, which in turn enhances monocarboxylate transporter 1 (MCT1)-mediated acetate uptake detectable by ^11^C-acetate PET^10^. We therefore tested whether ^18^F-THK5351 uptake is similarly increased under these conditions. In vivo PET imaging revealed markedly elevated ^18^F-THK5351 signals in the adenovirus-injected brain regions compared with contralateral, non-injected areas (Fig. 5b). To assess whether this increase reflected MAO-B activity, animals were treated with KDS2010, a potent and reversible MAO-B inhibitor^15^. ^18^F-THK5351 uptake was significantly reduced in KDS2010-treated rats relative to untreated controls (Fig. 5b, c), confirming that the elevated tracer signal originated from MAO-B upregulation in reactive astrocytes. These results demonstrate that ^18^F-THK5351 effectively detects MAO-B-mediated reactive astrogliosis in vivo, independent of amyloid or tau proteinopathy.

### Translation to human positron-emission tomography imaging: monoamine oxidase-B-dominant ¹⁸F-THK5351 signals in patients with corticobasal syndrome and Alzheimer’s disease

**Fig. 6:**
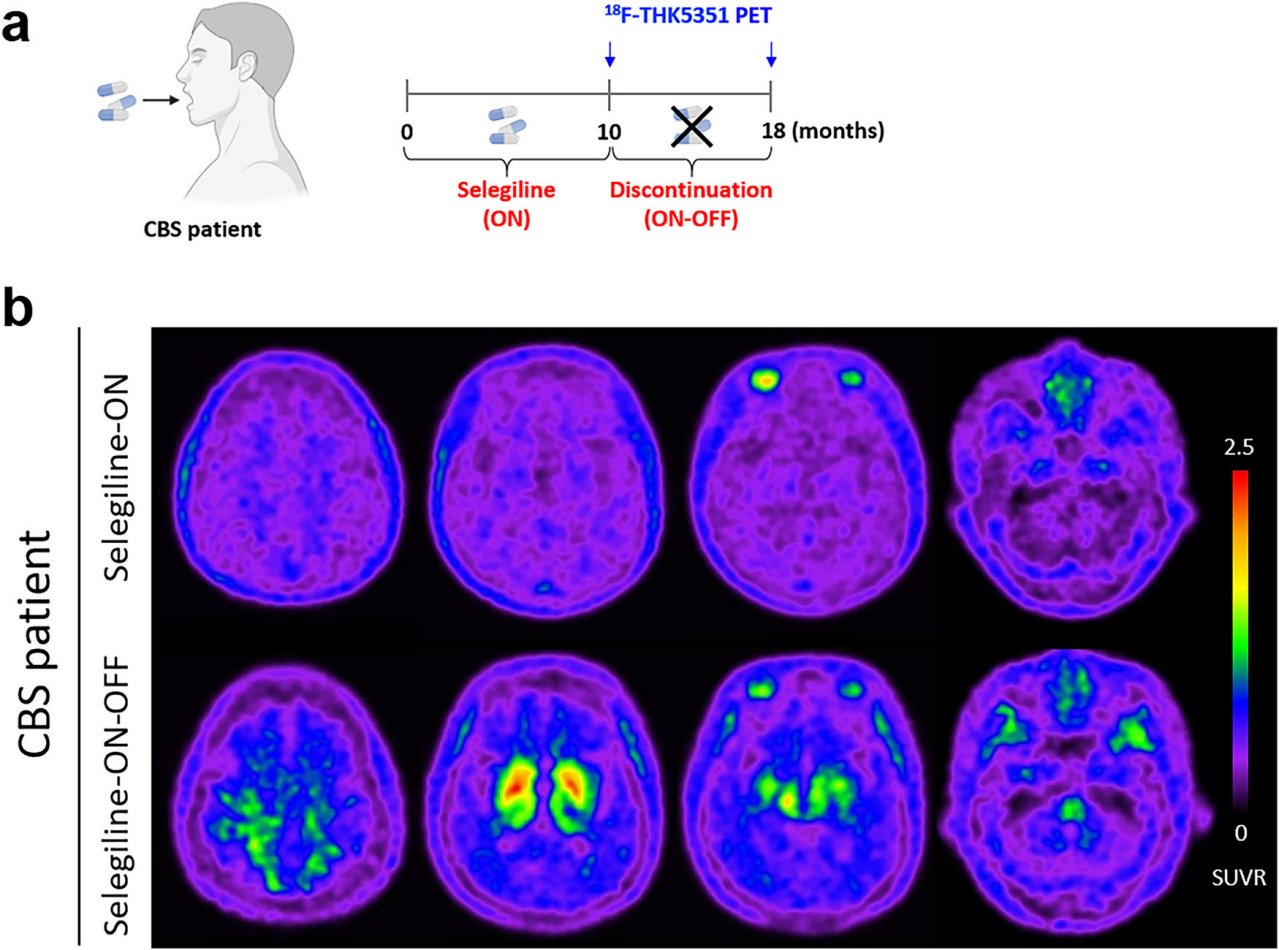
Monoamine oxidase-B-mediated THK5351 uptake in the brain of a patient with corticobasal syndrome. **a** Schematic description of positron-emission tomography (PET) imaging of a patient with corticobasal syndrome (CBS). **b** THK5351 uptake of the brain from the patient with CBS, during selegiline administration (up, Selegiline-ON) or after discontinuation (down, Selegiline-ON-OFF).

To translate our preclinical findings to human pathology, we examined ^18^F-THK5351 PET in a patient clinically diagnosed with corticobasal syndrome (CBS) with imaging and clinical features highly suggestive of corticobasal degeneration (CBD), a primary 4 R tauopathy (Fig. 6a). When scanned during oral selegiline administration (Selegiline-ON), ^18^F-THK5351 uptake was markedly reduced throughout the cortex and basal ganglia. After 8 months of selegiline discontinuation (Selegiline-ON-OFF), tracer uptake increased substantially, particularly in cortical and subcortical regions (Fig. 6b). These findings indicate that ^18^F-THK5351 binding in this patient is largely mediated by MAO-B activity, consistent with pharmacological blockade results in our preclinical models. Given the high likelihood of underlying CBD pathology, these data highlight the spatial coexistence of tau aggregates and MAO-B-associated astrogliosis, although their relative contributions cannot be distinguished visually on clinical PET images.

**Fig. 7:**
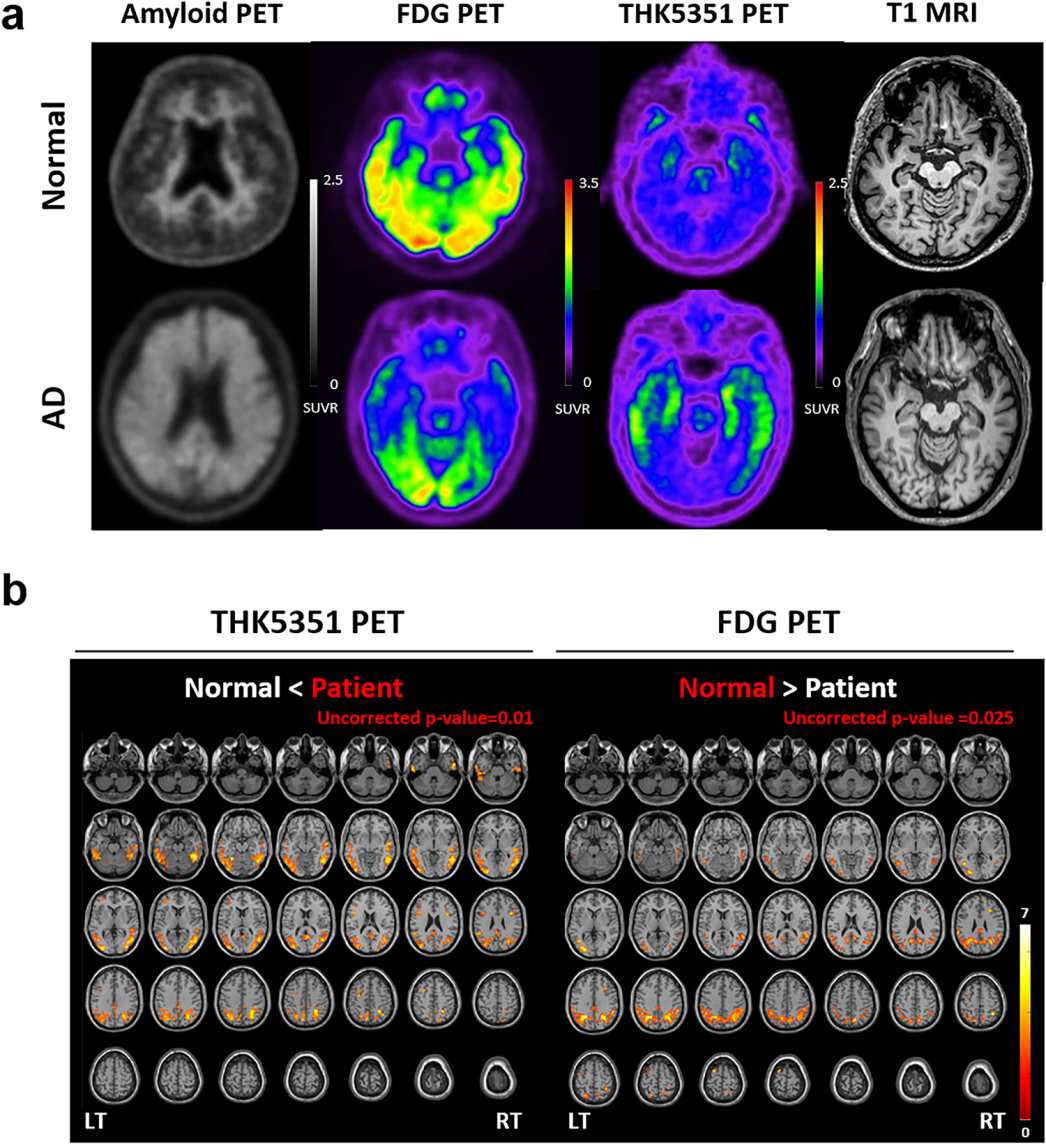
THK5351 uptake in the brain of patients with Alzheimer’s disease. **a** Amyloid, FDG and THK5351 positron-emission tomography (PET) images in the brain from both healthy control subjects and patients with Alzheimer’s disease (AD). Right: MRI in human brain with AD. **b** Unique imaging patterns obtained by contrasting normal control individuals with those diagnosed with AD.

We next compared ^18^F-THK5351 PET with amyloid and ^18^F-fluorodeoxyglucose (^18^F-FDG) PET in patients with AD and in healthy controls. Patients with AD exhibited widespread cortical amyloid deposition and regionally reduced glucose metabolism, particularly in the medial temporal and temporal neocortex (Fig. 7a). Although amyloid accumulation was diffusely increased, FDG reductions were more localized, illustrating that amyloid PET does not directly reflect neuronal dysfunction. In contrast, ^18^F-THK5351 uptake was significantly elevated in temporal lobe regions and showed a strong inverse correlation with ^18^F-FDG uptake (Fig. 7a, b), suggesting that MAO-B-associated reactive astrogliosis corresponds spatially to regions undergoing neurodegeneration.

## Discussion

In this study, we established a comprehensive, multi-modal validation framework to elucidate the molecular and cellular origins of ^18^F-THK5351 PET signals across molecular, tissue, animal and human levels. Our methodological approach addressed key limitations of prior THK5351 studies, which relied largely on indirect or correlative evidence of off-target binding. By integrating enzymatic assays, molecular docking, BLI, autoradiography, multiple rodent models and human PET imaging, we demonstrated that THK5351 binds directly to MAO-B with moderate potency and high selectivity over MAO-A, while exhibiting only weak affinity for tau, which was negligible on PET imaging in vivo. Collectively, these results converge on a consistent conclusion: in vivo ^18^F-THK5351 uptake primarily reflects MAO-B-associated reactive astrogliosis, with minor, often visually negligible, contributions from tau aggregates.

Enzyme inhibition assays provided a rapid and quantitative assessment of MAO-B selectivity, illustrating THK5351’s moderate potency (IC₅₀ = 0.78 μM) and negligible MAO-A inhibition. Although this assay offers practical advantages for screening and comparing tracers, it is constrained by its reliance on enzymatic activity rather than on direct binding, and by the micromolar concentration range used, which does not fully replicate the nanomolar tracer concentrations achieved in PET imaging.

BLI served as a direct, label-free biophysical validation, detecting reversible THK5351–MAO-B binding with an affinity consistent with enzymatic inhibition. However, immobilization effects and the use of micromolar concentrations limit the physiological relevance of the derived affinities, and signals may be influenced by mass transport, non-specific adsorption and protein orientation, particularly for disordered targets such as tau. Within these constraints, the data provide direct biophysical evidence that THK5351 interacts with MAO-B.

To complement these biochemical assays, in silico docking simulations were done using the Schrödinger program and SymDOCK. Schrödinger-based docking simulations localized THK5351 to the substrate funnel of MAO-B adjacent to FAD, providing structural insight and supplementary Gibbs free energy change after docking, into its selective binding. Although docking enables rapid, qualitative comparison of potential binding sites across multiple ligands, its predictive accuracy depends strongly on the chosen protein model, treatment of covalent interactions and scoring algorithms — factors that necessitate experimental validation.

SymDOCK modelling addressed this limitation by incorporating ligand–ligand van der Waals contributions, showing that MK6240 forms a stabilizing hydrogen bond with Lys353 and stronger stacking interactions than THK5351, whereas SMBT-1 completely lacks hydrogen bonding. Together, these results illustrate how integrating orthogonal methods can overcome the inherent weaknesses of any single assay — biochemical simplicity of enzyme assays, static assumptions of docking and structural incompleteness of monomer-based BLI — yielding a robust cross-validation pipeline.

At the tissue level, competitive autoradiography established a clear quantitative separation between MAO-B and tau contributions: approximately 80% of cortical ^18^F-THK5351 binding arose from MAO-B inhibition, whereas the remaining 20% reflected weak tau binding. This ratio was further supported by competition with MK6240 and differential displacement by MAO-B inhibitors. Importantly, although a small tau-derived component exists, it is unlikely to be visually discernible on clinical imaging, where background activity and signal attenuation obscure subtle differences — explaining why THK5351 PET patterns appear primarily astrocytic rather than fibrillar in patients.

In vivo rodent imaging reinforced these mechanistic insights. In APP/PS1 mice, ^18^F-THK5351 uptake paralleled MAO-B overexpression and was suppressed by selegiline, confirming tracer specificity. In contrast, localized overexpression of P301L-hTau induced robust tau pathology without affecting tracer uptake, whereas MAO-B knockout both suppressed THK5351 signals and reduced tau phosphorylation. These findings suggest that MAO-B is not only the principal molecular target of ^18^F-THK5351 but may also influence tau aggregation, possibly through astrocytic metabolic or oxidative stress pathways. The adenovirus-induced reactive astrogliosis model provided complementary validation by demonstrating that ^18^F-THK5351 can visualize MAO-B upregulation independent of proteinopathy and is reversible by selective MAO-B inhibition. Nevertheless, the use of acute viral models entails limitations — transient inflammation, exaggerated GFAP expression and immune confounds — that differ from the chronic, progressive gliosis of human disease.

Human studies extended these findings to the clinic. In postmortem tissue, ^3^H-THK5351 binding was displaced by MAO-B inhibitors but not by ligands for MAO-A or other receptors, confirming its selectivity. In AD cortex, residual signal after MAO-B blockade was modestly reduced by MK6240, consistent with minor tau binding. Clinically, ^18^F-THK5351 uptake in AD patients correlated inversely with FDG metabolism, mapping to neurodegeneration-associated regions. However, this inverse relationship should be interpreted with caution, as prior studies using flortaucipir have demonstrated that FDG PET can appear as a spatial “mirror image” of tau PET in individuals with substantial tau burden^63–66^. Given that ^18^F-THK5351 retains weak affinity for tau, it is possible that part of the observed inverse correlation with FDG reflects underlying tau pathology rather than purely reactive astrogliosis. Ideally, this distinction would be resolved by demonstrating, within the same individual, a pattern of low tau PET signal (e.g. MK6240), high THK5351 uptake and reduced FDG metabolism, which would more directly support an astrogliosis-driven mechanism. Although such multimodal imaging was not available in the present study, this limitation should be considered when interpreting the relationship between THK5351 and neurodegeneration. In a patient with CBS strongly suspected of underlying CBD, selegiline withdrawal led to a striking re-emergence of tracer uptake across the cortex and basal ganglia — direct evidence that MAO-B dominates in vivo signal formation.

From a methodological standpoint, this multi-scale validation strategy mitigates several classical limitations of PET tracer characterization. Small-animal PET provides a flexible, longitudinal approach for dynamic validation, yet differences in tracer pharmacokinetics, species metabolism and spatial resolution often confound quantitative interpretation. By pairing genetic and pharmacological perturbations across complementary models, we minimized these uncertainties and linked molecular binding to macroscopic imaging outcomes. Similarly, by triangulating biochemical, structural and imaging data, our framework enhances interpretability and reproducibility across experimental scales.

In summary, ^18^F-THK5351 predominantly reflects MAO-B-mediated reactive astrogliosis, with only weak contributions from tau aggregates that are unlikely to appear visually distinct on clinical imaging. Beyond refining the mechanistic understanding of this tracer, our study underscores the importance of multimodal, cross-scale validation pipelines in resolving the complex pharmacology of PET ligands. This approach not only clarifies the biological basis of historical THK5351 data but also provides a generalizable blueprint for evaluating dual-binding or pleiotropic PET tracers in neurodegenerative disease research.

## Availability of data and materials

All data generated or analysed during this study are included in this published article and its supplementary information files. If additional information is needed, please contact the corresponding author.

## Supplementary information

Supplementary Information
