# Supplementary material for: In-depth multimodal validation of 18F-THK5351 for imaging monoamine oxidase-B-mediated reactive astrogliosis in Alzheimer’s and related neurodegenerative diseases
Source: Exp Mol Med. 2026 Jul 1;58(7):2123–37. doi: 10.1038/s12276-026-01757-5 (PMC13434134; doi:10.1038/s12276-026-01757-5)
Supplement: Supplementary file 1 [file 12276_2026_1757_MOESM1_ESM.pdf]

Supplementary Information

In-depth Multimodal Validation of ^{18}F-THK5351 for Imaging MAO-B-mediated Reactive Astrogliosis in Alzheimer's and Related Neurodegenerative Diseases

Heejung Chun[1,2,9*], Wongu Youn[3,9], Hyunkeong Lim[4,9], Ryuichi Harada[5,6], Hae Young Ko[4], Jiyoung Yoon[1,2], Soo-Jin Oh[7], Philipp Seemann[8], Joseph G. Pepe[8], Brian K. Shoichet[8], Shozo Furumoto[6], Nobuyuki Okamura[5,6], Mijin Yun[4*], C Justin Lee[3*]

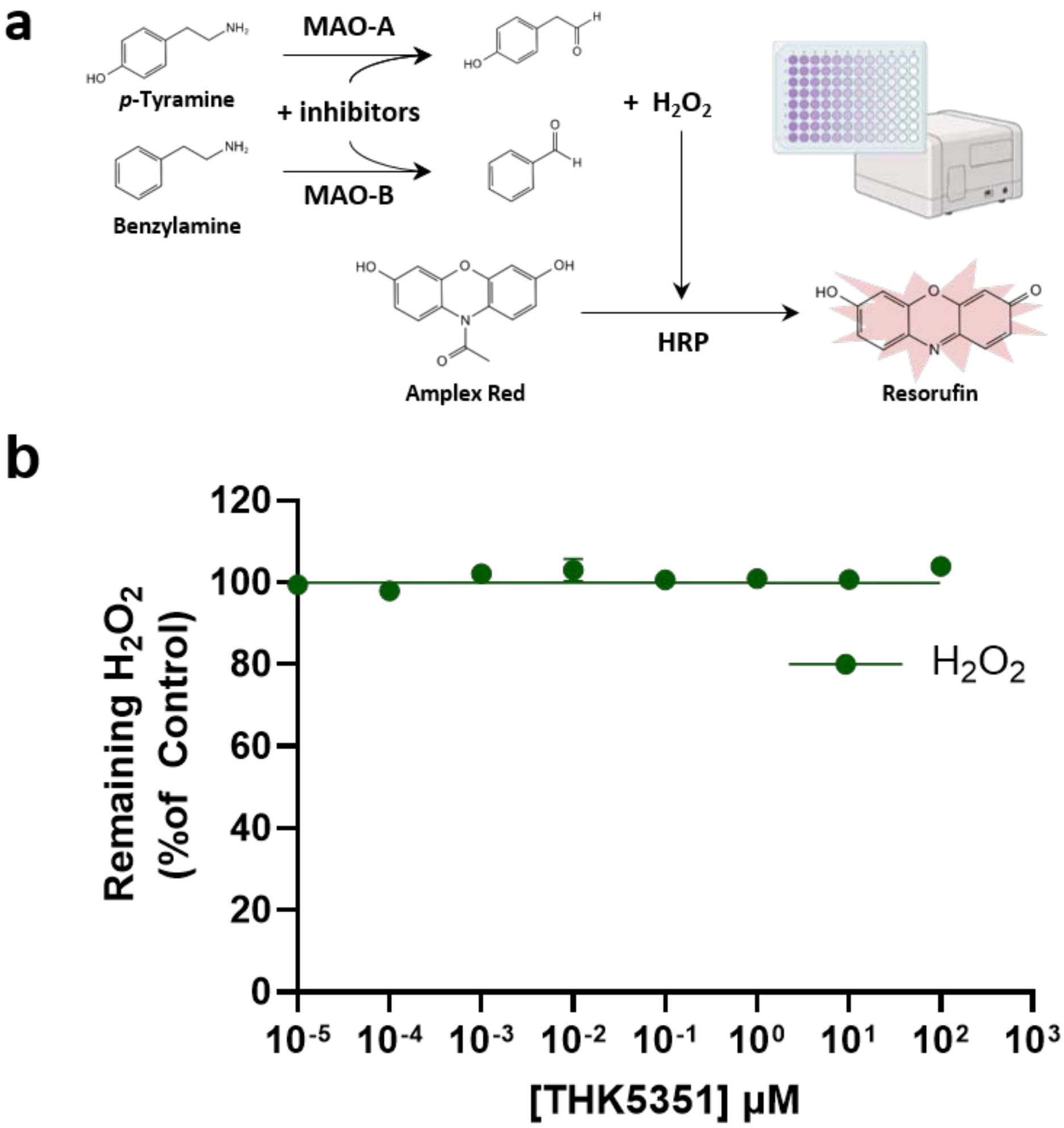

Supplementary Figure 1. Enzyme activity assay of THK5351. a. Schematic description of MAO-A and MAO-B enzyme assay. **b**. Amplex Red H_2O_2 assay with THK5351. Data are presented as the mean ± s.e.m.

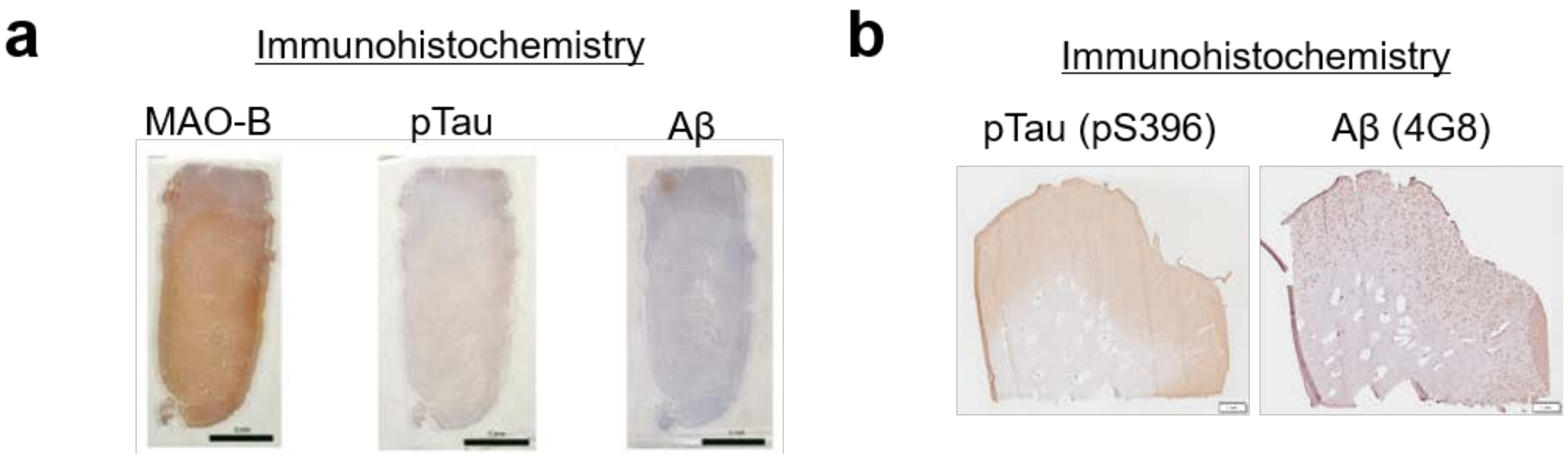

Supplementary Figure 2. IHC images of human brain section. a. IHC images of MAO-B, pTau and Aβ in human basal ganglia tissue. **b.** IHC images of pTau(S396) and Aβ in human AD brain tissue.

a

b

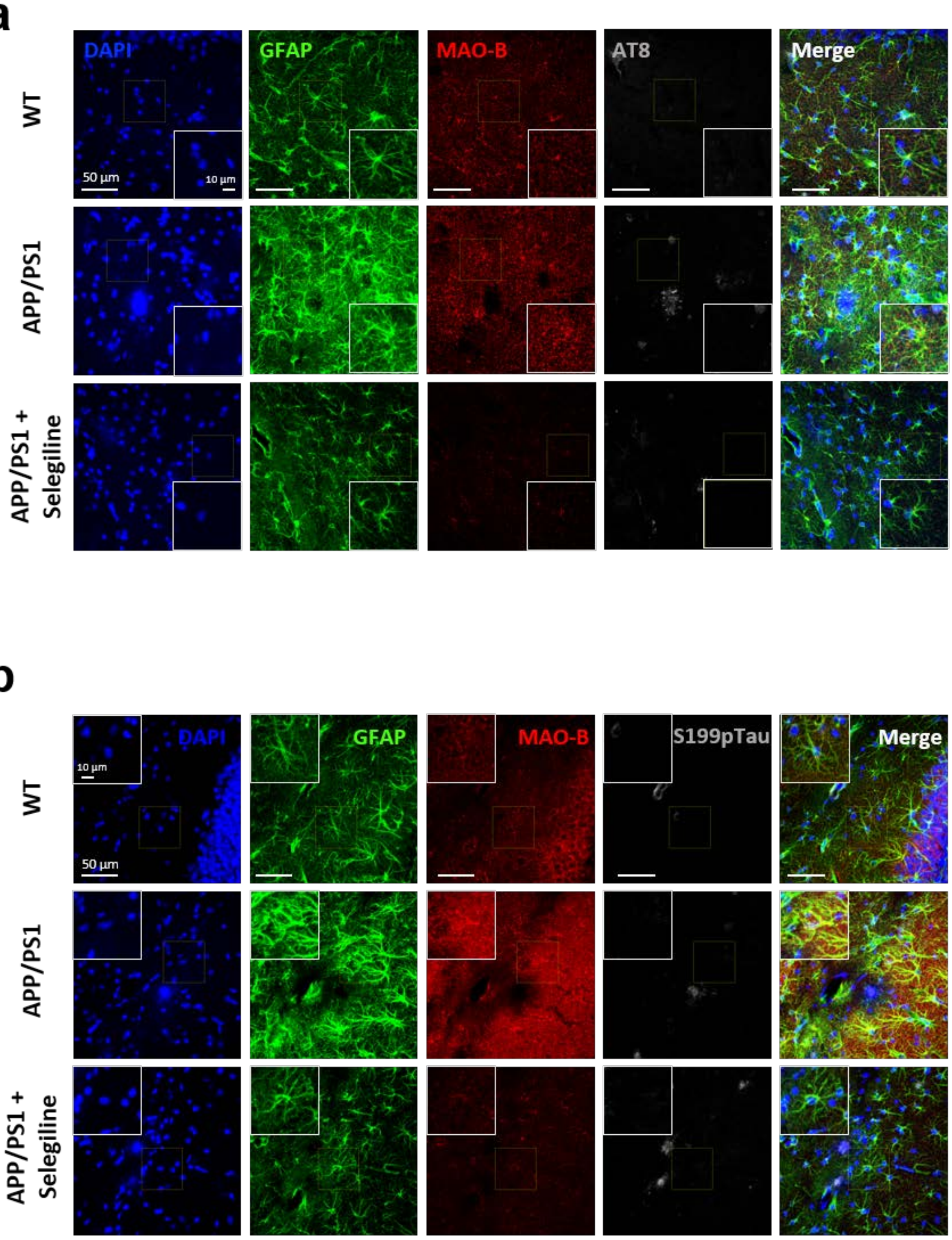

Supplementary Figure 3. Long-term treatment of selegiline efficiently inhibits the reactivity of astrocytes and the expression of MAO-B in APP/PS1 mice. a, b. Representative IHC images of GFAP, MAO-B and pTau(AT8 (**a**), S199 (**b**)) in selegiline-treated APP/PS1 mice brain.

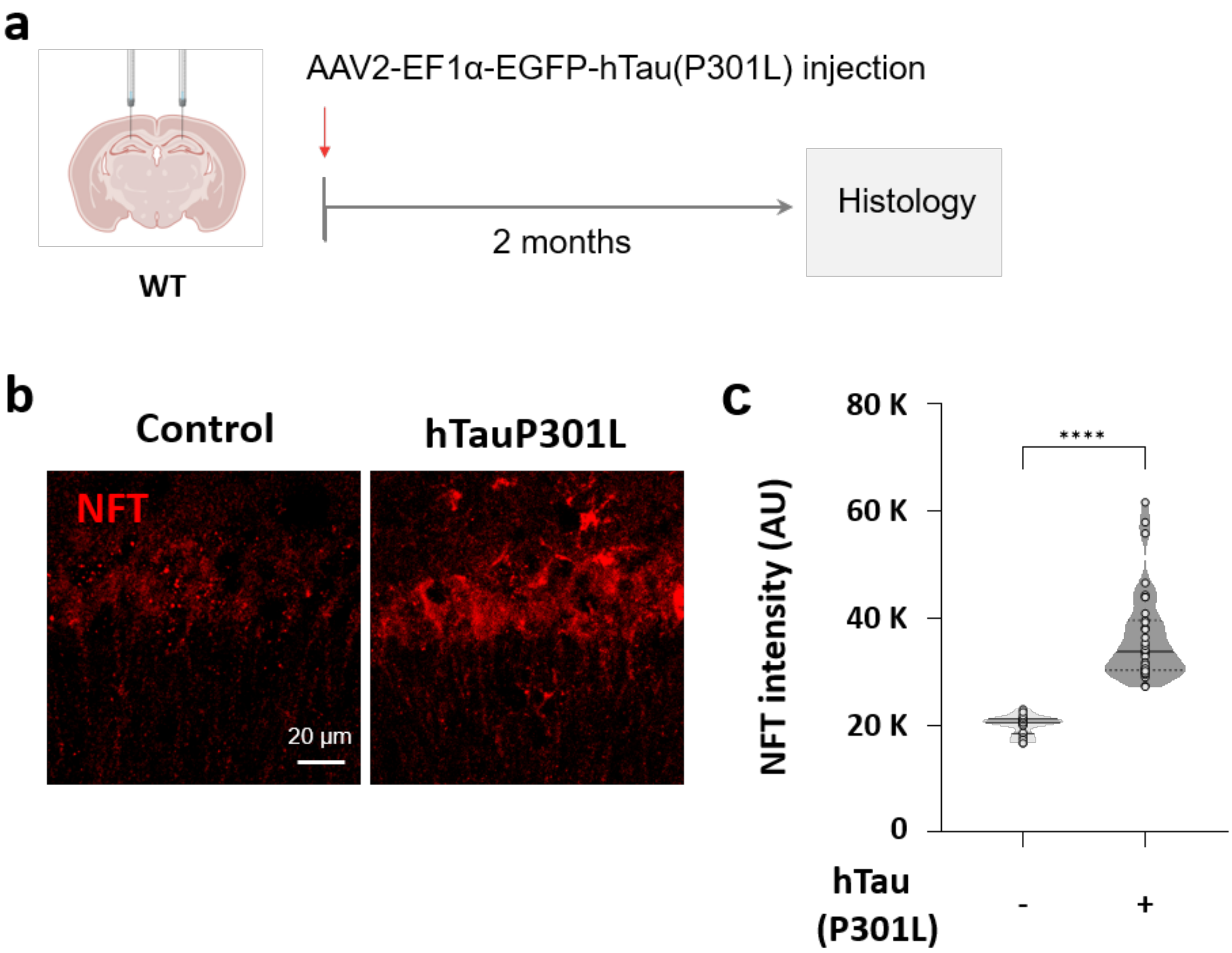

Supplementary Figure 4. Overexpression of mutated tau protein, hTauP301L, in hippocampal CA1 region induces neurofibrillary tangle formation. a. Schematic description of hTauP301L virus injection. **b.** IHC image of NFT in hTauP301L virus injected mice or control mice hippocampus. **c.** Quantification of NFT intensity. Data are shown as violin plots with individual points, median, and interquartile range (25th-75th) indicated. ****P < 0.0001.
